# Sodium-glucose cotransporter-2 inhibitors and incidence of atrial fibrillation in older adults with type 2 diabetes: a retrospective cohort analysis

**DOI:** 10.3389/fphar.2024.1379251

**Published:** 2024-05-23

**Authors:** Yujia Li, Huilin Tang, Yi Guo, Hui Shao, Stephen E. Kimmel, Jiang Bian, Desmond A. Schatz, Jingchuan Guo

**Affiliations:** ^1^ Department of Pharmaceutical Outcomes and Policy, University of Florida College of Pharmacy, Gainesville, FL, United States; ^2^ Department of Health Outcomes and Biomedical Informatics, University of Florida College of Medicine, Gainesville, FL, United States; ^3^ Hubert Department of Global Health, Rollins School of Public Health, Emory University, Atlanta, GA, United States; ^4^ Department of Family and Preventive Medicine, School of Medicine, Emory University, Atlanta, GA, United States; ^5^ Department of Epidemiology, University of Florida College of Public Health and Health Professions and College of Medicine, Gainesville, FL, United States; ^6^ Department of Pediatrics, University of Florida College of Medicine, Gainesville, FL, United States

**Keywords:** type 2 diabetes, atrial fibrillation, SGLT2 inhibitors, retrospective cohort, comparative effectiveness

## Abstract

**Objectives:**

To investigate the risk of atrial fibrillation (AF) with sodium-glucose cotransporter-2 inhibitors (SGLT2is) compared to dipeptidyl peptidase-4 inhibitor (DPP4i) use in older US adults and across diverse subgroups.

**Methods:**

We conducted a retrospective cohort analysis using claims data from 15% random samples of Medicare fee-for-service beneficiaries. Patients were adults with type 2 diabetes (T2D), no preexisting AF, and were newly initiated on SGLT2i or DPP4i. The outcome was the first incident AF. Inverse probability treatment weighting (IPTW) was used to balance the baseline covariates between the treatment groups including sociodemographics, comorbidities, and co-medications. Cox regression models were used to assess the effect of SGLT2i compared to DPP4i on incident AF.

**Results:**

Of the 97,436 eligible individuals (mean age 71.2 ± 9.8 years, 54.6% women), 1.01% (n = 983) had incident AF over a median follow-up of 361 days. The adjusted incidence rate was 8.39 (95% CI: 6.67–9.99) and 11.70 (95% CI: 10.9–12.55) per 1,000 person-years in the SGLT2i and DPP4i groups, respectively. SGLT2is were associated with a significantly lower risk of incident AF (HR 0.73; 95% CI, 0.57 to 0.91; *p* = 0.01) than DPP4is. The risk reduction of incident AF was significant in non-Hispanic White individuals and subgroups with existing atherosclerotic cardiovascular diseases and chronic kidney disease.

**Conclusion:**

Compared to the use of DPP4i, that of SGLT2i was associated with a lower risk of AF in patients with T2D. Our findings contribute to the real-world evidence regarding the effectiveness of SGLT2i in preventing AF and support a tailored therapeutic approach to optimize treatment selection based on individual characteristics.

## 1 Introduction

Type 2 diabetes (T2D) is an increasingly prevalent condition and has become a public health concern with population aging. The metabolic changes associated with diabetes, such as glycemic fluctuations and induction of oxidative stress and inflammation, could result in structural, mechanical, autonomic, and electrical remodeling of the atrium, increasing the risk of arrhythmias (Wang et al., 2019). Atrial fibrillation (AF) is the most common sustained arrhythmia, with a rising incidence and prevalence in T2D patients (Bell and Goncalves, 2019). The comorbidity of T2D and AF is associated with adverse cerebrovascular and cardiovascular outcomes and an increased mortality rate (Karayiannides et al., 2018; Bell and Goncalves, 2019). Therefore, preventing and managing AF in T2D patients is crucial in managing subsequent ischemic stroke and cardiovascular morbidities associated with these two conditions.

Several glucose-lowering drugs (GLDs), such as metformin and certain thiazolidinediones (pioglitazone), have been suggested to reduce atrial remodeling and lower the risk of AF among T2D patients (Wang et al., 2019). Sodium-glucose cotransporter-2 (SGLT2) inhibitors, a novel class of oral GLD, have demonstrated significant efficacy in glycemic control, weight loss, and blood pressure (Bolinder et al., 2012; Shyangdan et al., 2016), as well as effectiveness in reducing major atherosclerotic cardiovascular events and renal function decline in clinical trials (Zinman et al., 2015; Neal et al., 2017; McGuire et al., 2021) and observational studies using real-world data (McGuire et al., 2021). Despite their safety and effectiveness profile, the role of SGLT2 inhibitors in the primary or secondary prevention of AF is inconclusive. Mechanistic studies show that SGLT2 inhibitors could inhibit the sodium–hydrogen exchange in cardiac myocytes, which may ameliorate myocardial remodeling and, thus, reduce the risk of AF (Arow et al., 2020; Cowie and Fisher, 2020). Nevertheless, clinical studies examining the association between SGLT2 inhibitors and AF have yielded conflicting results. Two cardiovascular safety trials, the EMPA-REG OUTCOME trial (empagliflozin) and CANVAS (canagliflozin), did not report any significant difference in AF incidence for T2D patients on SGLT2 inhibitors compared with placebo (Zinman et al., 2015; Neal et al., 2017). A *post hoc* analysis of the DECLARE-TIMI 58 trial suggested that dapagliflozin, compared with placebo, was associated with a decreased risk of AF or atrial flutter (AFL) in patients with T2D (Zelniker et al., 2020), but this endpoint was not prespecified, and the trial only included T2D patients with multiple risk factors for or established cardiovascular conditions. Moreover, real-world evidence is scarce regarding the effect of SGLT2i on the risk of AF, with most of the results from Asian or Northern European populations (Persson et al., 2018; Ling et al., 2020; Chan et al., 2022; Lee et al., 2022), which may not be generalizable to the US population. Therefore, further research is needed to determine the potential role of SGLT2 inhibitors in preventing AF among patients with T2D across a diverse population.

The objective of this study is to address this research gap by assessing the association between SGLT2 inhibitor use and AF risk in a nationally representative sample of T2D patients in the United States and determine any effect variation across sex, race/ethnicity, and baseline comorbidities. We considered the dipeptidyl peptidase-4 (DPP4) inhibitors a well-suited comparator for examining the effectiveness of another GLD because of their wide use in T2D treatment (American Diabetes Association, 2021) and the neutral effect on cardiovascular outcomes (Scirica et al., 2013; Green et al., 2015). We aimed to provide a comprehensive evaluation of the potential impact of SGLT2 inhibitors on AF risk, thereby suggesting a more precise therapeutic approach for patients with T2D.

## 2 Materials and methods

### 2.1 Data source

This was a retrospective cohort study assessing the comparative effectiveness of SGLT2 inhibitors versus DPP4 inhibitors in association with the risk of incident AF among individuals with T2D using claims data from 2017–2018 of 15% random samples of national Medicare fee-for-service beneficiaries. Medicare is the largest health insurance provider for adults ≥ 65 years in the United States, covering 98% of this population (Chronic conditions data warehouse, 2022b). The Medicare claims database contains billing records for inpatient and outpatient encounters and dispensed prescription drugs. It also provides beneficiary-level information on the sociodemographic characteristics and health-plan enrollment status. It is certified as de-identified; therefore, this study was considered exempt from review by the Institutional Review Board of the University of Florida.

### 2.2 Study participants

**FIGURE 1:**
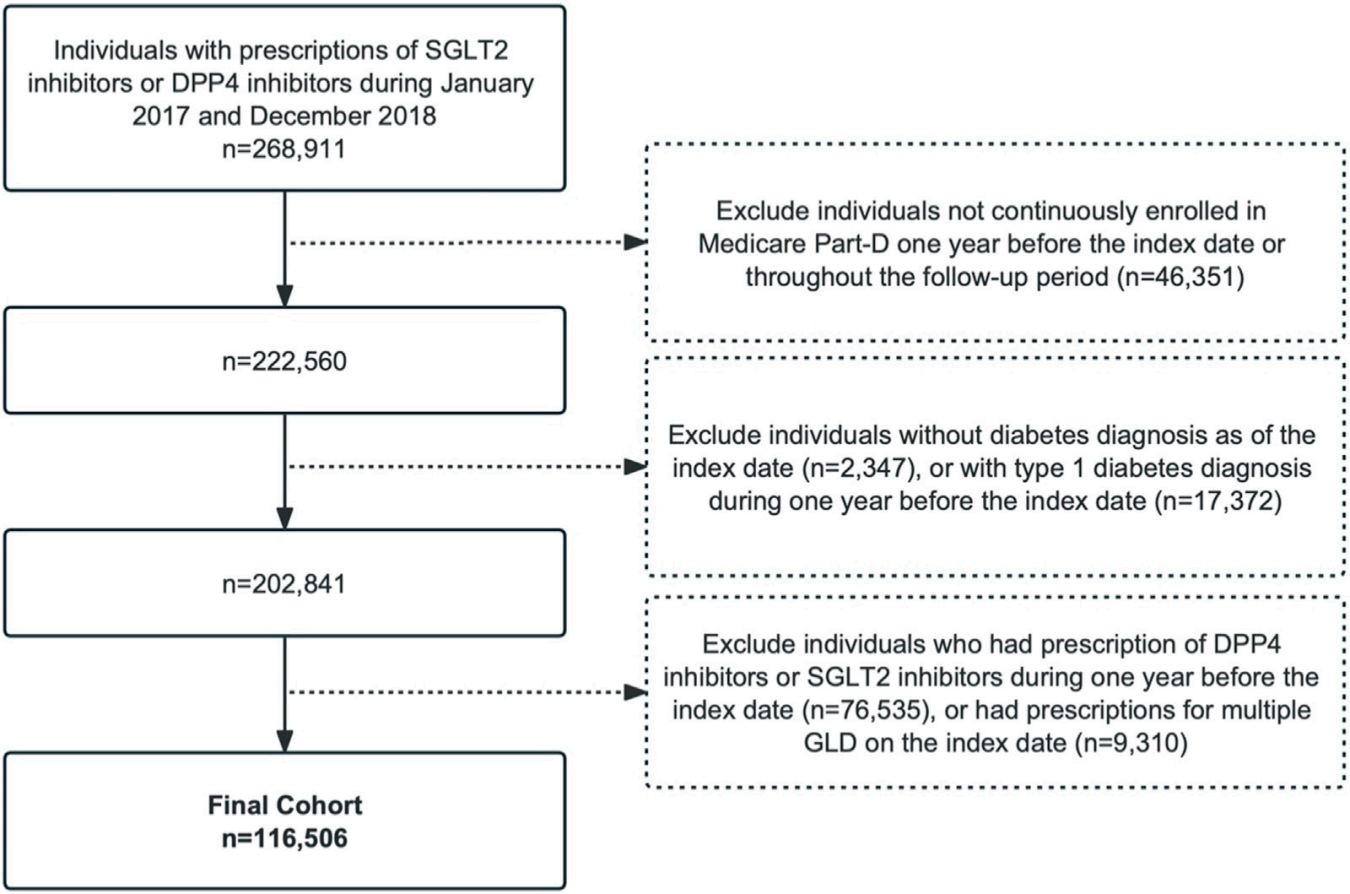
Patient flowchart. SGLT2, sodium-glucose co-transporter-2; DPP4, dipeptidyl peptidase-4; GLDs, glucose-lowering drugs.

We identified individuals with T2D following the initiation of an SGLT2 inhibitor (canagliflozin, dapagliflozin, or empagliflozin) or DPP4 inhibitor (sitagliptin, saxagliptin, alogliptin, or linagliptin) from 1 January 2017 to 31 December 2018. The index date was the day of initiation of an SGLT2 inhibitor or DPP4 inhibitor, which was defined as the date of the first prescription of the drug with no use of either in the prior year (i.e., the baseline year). We included individuals with a diagnosis of T2D preceding the index date using the International Classification of Diseases, Ninth Revision, Clinical Modification (ICD-9 CM) or Tenth Revision (ICD-10 CM). We excluded individuals who had a type 1 diabetes diagnosis in inpatient or outpatient claims over the study period. Individuals without continuous enrollment in the Part D fee-for-service plan in the baseline year or during the follow-up period were excluded to ensure that we captured the complete medical and prescription information of the study cohort. We also excluded individuals with preexisting AF diagnoses to obtain incident AF cases during the follow-up. Patients were followed-up until the occurrence of the outcome, death, switching to another therapeutic class (i.e., SGLT2 inhibitor users initiating a DPP4 inhibitor or *vice versa*), or 31 December 2018, whichever occurred first. The flowchart of the study design and patient enrollment is shown in Figure 1.

### 2.3 Outcomes

The outcome was the time from the index date to the first incident AF event during the follow-up period. Incident AF was defined as having a diagnosis in an inpatient or outpatient claim with diagnosis codes ICD-9 427.31 or ICD-10 I48, respectively, which have been validated in previous studies (positive predicted values [PPV] = 88%) (Yao et al., 2019).

### 2.4 Exposures and covariates

The exposure of interest was the initiation of an SGLT2 inhibitor, compared with the initiation of a DPP4 inhibitor.

We included a comprehensive set of covariates in our analysis, which was selected based on previous literature (Patorno et al., 2018), including sociodemographics (age, sex, race and ethnicity, Medicare–Medicaid dual eligibility, and enrollment of low-income subsidy); duration of diabetes, chronic comorbidities (acute myocardial infarction, Alzheimer’s disease, dementia, cataract, chronic kidney disease (CKD), chronic obstructive pulmonary disease, heart failure (HF), glaucoma, hip or pelvic fracture, ischemic heart disease, vascular heart disease, depression, osteoporosis, rheumatoid arthritis or osteoarthritis, stroke, acquired hyperthyroidism, and cancers); and baseline medications (use of antidiabetic medications including insulins, metformin, sulfonylureas, glucagon-like peptide-1 receptor agonists, thiazolidinediones, meglitinides, alpha-glucosidase inhibitors, and amylin analogs and use of other medications including antidepressants, opioids, beta-receptor blockers, angiotensin-converting enzyme inhibitors, calcium channel blockers, angiotensin-II receptor blockers and diuretics, statins, nonsteroidal anti-inflammatory drugs, oral steroids, antiplatelets, anticoagulants, and anti-arrhythmic drugs). The duration of diabetes was a *post hoc* variable generated by the time from the first-ever date of diabetes diagnosis in Medicare claims to the index date and was used as a proxy for the severity of diabetes. The patients’ covariates such as sociodemographics and comorbidities were measured during the 1-year baseline period (Chronic conditions data warehouse, 2022a). The baseline medication information was collected during the 6-month period prior to the index date.

### 2.5 Statistical analyses

Descriptive statistics were reported to describe the baseline characteristics of patients with T2D initiating SGLT2 or DPP4 inhibitors. Means and standard deviations (SD) were reported for continuous variables, and percentages were reported for categorical variables.

We employed stabilized inverse probability of treatment weighting (IPTW) to adjust for potential imbalances of confounding factors between the two treatment groups. IPTW, computed based on the propensity scores (PSs), was used to create a pseudo-population, in that the measured baseline covariates are equally distributed across treatment groups (Austin and Stuart, 2015). We calculated the PS using multivariate logistic regressions adjusting for 52 covariates, including sociodemographic, chronic comorbidities, and baseline medications, and calculated the stabilized IPTW according to the PS (Stürmer et al., 2010). Through the asymmetrical trimming principle, we excluded individuals with PSs below the 2.5th percentile of the PS distribution for SGLT2 inhibitors or above the 97.5th percentile of the DPP4 inhibitors’ PS distribution to improve comparability (Stürmer et al., 2010). The balance in covariates between the two groups was assessed using standardized mean difference (SMD) before and after the weighting, with an absolute value of 0.1 or less indicating a good balance of covariates between the two treatment groups (Austin, 2009).

We used the Cox proportional hazard regression model to compare the effectiveness of SGLT2 inhibitors compared to DPP4 inhibitors on the risk of AF after incorporating IPTW. The analyses were based on the intension-to-treat method, which means the patients were analyzed according to the drug they started with, regardless of whether they switched their drugs or not. In exploratory hypothesis-generating analyses, we further studied the effectiveness of SGLT2 inhibitors compared to DPP4 inhibitors on the risk of AF in predefined subgroups of sex, race/ethnicity (non-Hispanic White, non-Hispanic Black, Hispanic, and others), with and without atherosclerotic cardiovascular diseases (ASCVD, defined as patients with myocardial infarction, ischemic heart disease, and stroke), and CKD and HF at baseline.

All analyses were performed using SAS 9.4 (SAS Institute, Cary, NC). We used an alpha-level of 0.05 to evaluate statistical significance.

## 3 Results

We identified 97,436 eligible Medicare beneficiaries with T2D and initiated an SGLT2 inhibitor or DPP4 inhibitor during 2017–2018, of which 80,949 (83.1%) were DPP4 inhibitor users and 16,487 (16.9%) were SGLT2 inhibitor users. The study cohort was followed-up for a median of 361 (interquartile range [IQR] 202–467) days.

Table 1 summarizes the sociodemographic, baseline comorbidities, and medications of the two treatment groups. Before applying IPTW, SGLT2 inhibitor users were younger and mostly men and non-Hispanic White than DPP4 inhibitor users. SGLT2 inhibitor users had a lower prevalence of chronic kidney disease, heart failure, and a history of stroke, and they were more likely to have used insulin, metformin, and glucagon-like peptide-1 (GLP1) agonists in the baseline period when compared to DPP4 inhibitor users. We observed a total of 983 (1.0%) incident AF events during the follow-up period, with 124 (0.8%) and 849 (1.1%) incident AF events occurring in the SGLT2 inhibitor and DPP4 inhibitor users, respectively. The crude incidence rate of AF was 11.28 (95% confidence interval [CI], 10.60–11.99) events per 1,000 person-years (PY) in the overall study cohort. The crude incidence rate of AF was 8.37 (95% CI, 7.01–9.99) and 11.79 (95% CI, 11.03–12.61) events per 1,000 PY for SGLT2 inhibitor users and DPP4 inhibitor users, respectively.

**TABLE 1:** Patient characteristics before inverse propensity weighting by treatment, and standardized median differences before and after inverse probability treatment weighting.

	DPP4 inhibitor user (n = 80,949)	SGLT2 inhibitor user (n = 16,487)	Absolute SMD before IPTW	Absolute SMD after IPTW
Age, mean (SD)	72.20 (10.14)	67.09 (9.65)	0.516	0.053
Female, %	56.23	47.36	0.178	0.014
Race/ethnicity, %				
Non-Hispanic White	61.60	72.09	0.221	0.045
Non-Hispanic Black	14.13	10.11		
Hispanic	15.50	11.17		
Others	8.77	6.62		
Medicare and Medicaid dual eligibility, %	42.67	35.44	0.149	0.063
Diabetes duration, year, mean (SD)	8.73 (5.21)	6.68 (4.55)	0.418	0.012
Baseline comorbidities, %				
Acute myocardial infarction	5.46	3.82	0.078	0.025
Alzheimer’s disease	6.78	2.16	0.225	0.018
Dementia	17.39	7.79	0.293	0
Cataract	64.33	51.77	0.260	0.017
Chronic kidney disease	72.28	69.55	0.061	0.019
COPD	30.04	24.99	0.113	0.021
Heart failure and non-ischemic heart disease	31.32	21.31	0.229	0.005
Glaucoma	29.79	23.29	0.148	0.005
Hip or pelvic fracture	2.55	1.06	0.112	0.004
Ischemic heart disease	58.70	51.10	0.153	0.017
Depression	43.13	41.60	0.031	0.009
Osteoporosis	19.55	11.12	0.236	0.009
Rheumatoid arthritis or osteoarthritis	64.89	58.12	0.138	0.005
Stroke	16.94	11.01	0.171	0.004
Breast cancer	5.22	3.73	0.072	0.009
Colorectal cancer	2.79	1.72	0.073	0.016
Prostate cancer	5.37	4.47	0.043	0.013
Lung cancer	1.28	0.78	0.050	0.011
Endometrial cancer	1.24	0.75	0.049	0.006
Anemia	66.11	50.02	0.330	0.008
Asthma	19.05	18.06	0.026	0.009
Hyperlipidemia	95.89	95.36	0.027	0.013
Benign prostate hyperplasia	21.86	20.08	0.044	0.005
Hypertension	96.64	94.77	0.093	0.010
Acquired hypothyroidism	33.45	30.80	0.060	0.014
Vascular heart disease	24.95	19.48	0.132	0.003
Baseline co-medications, %				
Antidepressants	31.59	34.80	0.069	0.007
Opioids	21.02	25.17	0.099	0.002
Beta-receptor blockers	45.10	39.90	0.105	0.020
Statin	76.82	77.71	0.022	0.008
ACE inhibitors	35.53	36.92	0.030	0.017
Calcium channel blockers	34.09	26.76	0.160	0.006
ARB	35.49	32.36	0.067	0.008
Diuretics	28.32	22.73	0.128	0.013
Antipsychotics	4.37	4.95	0.027	0.003
NSAID	19.24	20.72	0.037	0.024
Oral steroids	26.79	25.54	0.028	0.005
Antiplatelets	2.23	1.84	0.028	0.019
Aldosterone receptor antagonists	3.23	3.03	0.012	0.003
Anticoagulants	2.67	2.63	0.003	0.004
Antiarrhythmic drugs	1.02	0.82	0.021	0.016
Glucose-lowering drugs at baseline, %				
Insulin	21.78	32.26	0.238	0.008
Metformin	42.00	62.02	0.409	0.007
Sulfonylureas	38.07	34.96	0.064	0.041
GLP1 receptor agonists	3.05	22.04	0.599	0.003
Thiazolidinediones	7.66	8.57	0.034	0.008
Meglitinides	2.26	1.64	0.045	0.010
AGI	0.62	0.56	0.009	0.002
Amylin analogs	0.01	0.03	0.018	0.001

Data are expressed as mean [standard deviation (SD)] or as percentage %.

SGLT2, sodium-glucose co-transporter-2; DPP4, dipeptidyl peptidase-4; SMD, standardized mean difference; IPTW, inverse probability treatment weighting; COPD, chronic obstructive pulmonary disease; ACE, angiotensin-converting enzyme; ARB, angiotensin receptor blocker; NSAID, nonsteroidal anti-inflammatory drug; GLP1, glucagon-like peptide-1; AGI, alpha glucosidase inhibitor.

**FIGURE 2:**
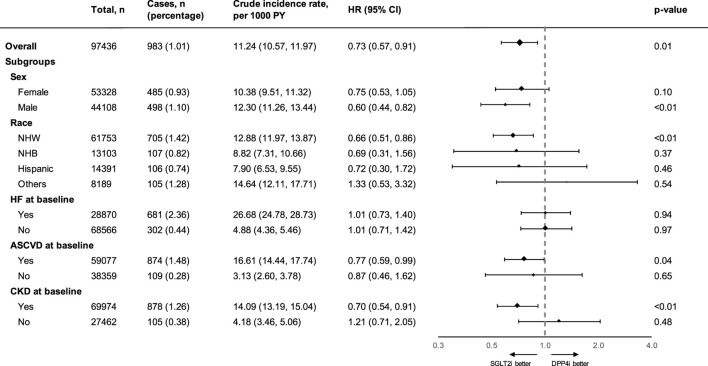
Summary of the effect of SGLT-2 inhibitors on atrial fibrillation outcomes compared with that of DPP-4 inhibitors in different subgroups. PY, person-years; HR, hazard ratio; CI, confidence interval; NHW, non-Hispanic White; NHB, non-Hispanic Black; HF, heart failure and non-ischemic heart disease; ASCVD, atherosclerotic cardiovascular disease; CKD, chronic kidney disease; SGLT2i, sodium glucose co-transporter-2 inhibitor; DPP4i, dipeptidyl peptidase-4 inhibitor. Atherosclerotic cardiovascular disease was defined as occurrences of myocardial infarction, ischemic heart disease, or stroke.

The two treatment groups were well-balanced in baseline covariates after IPTW (with all absolute values of SMD <0.1, Table 1). The adjusted incidence rate was 8.39 (95% CI, 6.67–9.99) events per 1,000 PY in the SGLT2 inhibitor group and 11.70 (95% CI, 10.9–12.55) per 1,000 PY in the DPP4 inhibitor group. SGLT2 inhibitor use was associated with a significantly lower risk of incident AF compared with DPP4 inhibitor use after inverse probability of treatment weighting (HR, 0.73; 95% CI, 0.57 to 0.91; *p* = 0.01; Supplementary Figure S1). The use of SGLT2 inhibitors was associated with a significantly lower risk of incident AF compared with the use of DPP4 inhibitors among men (HR, 0.60; 95% CI, 0.44 to 0.82; *p* < 0.01), but not in women (HR, 0.75; 95% CI, 0.53 to 1.05; *p* = 0.10). In the analyses by race and ethnicity, SGLT2 inhibitors appeared to have the most significant benefits on AF risk reduction in non-Hispanic White individuals (HR, 0.66; 95% CI, 0.51 to 0.86; *p* < 0.01). The effect was not significant in other race/ethnicity subgroups. In addition, among patients with established CVD and CKD, the observed benefit of SGLT2 inhibitor use on AF risk reduction was particularly apparent than in those without these conditions. However, the effect of SGLT2 inhibitors on AF risk reduction was not significant for subgroups both with and without HF at the baseline (Figure 2). In the subgroup analysis that stratified patients by concomitant use with other GLDs, the effect of SGLT2 inhibitors on AF risk reduction was observed among patients who had not used thiazolidinediones and/or sulfonylureas in the baseline. However, the effect was not significant among patients who had or had never used metformin (Supplementary Table S1).

## 4 Discussion

Our study compared the risk of incident AF between SGLT2 inhibitor users and DDP4 inhibitor users in an older US population using a nationally represented sample from Medicare claims data. Our results have demonstrated that the use of SGLT2 inhibitors compared to DPP4 inhibitors was associated with a significant reduction in the risk of incident AF among patients with T2D. Furthermore, in the exploratory analyses, we identified that the benefit of SGLT2 inhibitors on AF risk reduction was significant in men and the non-Hispanic White group, as well as among the most susceptible individuals with established ASCVD or CKD.

The SGLT2 inhibitor is the newest class of oral GLD agents that inhibits absorption of glucose by the proximal tubules of the kidney, resulting in glycosuria (Cowie and Fisher, 2020). Several potential mechanisms have been proposed for explaining the protective effect of SGLT2 inhibitors against AF. Preclinical data showed that SGLT2 inhibitors inhibit sodium–hydrogen exchange in cardiac myocytes, which may ameliorate myocardial hypertrophy, fibrosis, remodeling, and heart failure (Arow et al., 2020) and, therefore, lower the risk of AF. Moreover, SGLT2 inhibitors can inhibit sympathetic overdrive, which plays an important role in the development of AF (Herat et al., 2020). In addition, recent studies suggested that SGLT2 inhibitors had more favorable pleiotropic effects on HbA1c level, body weight, and systolic blood pressures when compared with DPP4 inhibitors, which may result in further arterial dilation and improved atrial remodeling and, thus, reduce the occurrence of AF (Schotten et al., 2011; Kim et al., 2019).

Until now, there has been limited evidence on the preventive effect of SGLT2 inhibitors against AF from randomized clinical trials. We identified a *post hoc* analysis from the DECLARE-TIMI 58 trial, which showed that dapagliflozin was associated with a reduced risk of AF/AFL events by 19% (95% CI, 5%–32%) (Zelniker et al., 2020). However, the AF/AFL events from this trial were not prespecified and identified based on an adverse event reported by site investigators, and the results were likely to be biased toward null. Furthermore, most of the trial participants had established cardiovascular conditions, which is different from our cohort.

Real-world evidence of the effect of SGLT2 inhibitors on the risk of AF remains limited. The CVD-REAL Nordic, an observational cohort study, failed to identify any difference in AF risk between dapagliflozin and DPP4 inhibitors (HR: 0.92; 95% CI, 0.76–1.12) (Persson et al., 2018). The discrepancies between our findings and those of the CVD‐REAL Nordic study might be explained by the different characteristics of the study populations. Compared to our Medicare cohort, the CVD-REAL Nordic participants were much younger (mean age: 61 years) and predominantly Caucasian. In addition, about 7% of the CVD-REAL Nordic study participants reported having AF at the baseline; it is thus unclear how the researchers distinguish incident AF from prevalent AF in the identification of the outcome. On the other hand, few studies reported a lower AF risk associated with SGLT2 inhibitors in East Asian populations. Chan et al. reported that SGLT2 inhibitors were associated with a lower risk of new-onset AF in patients with T2D compared with DPP4 inhibitors (HR, 0.90; 95% CI, 0.84–0.96) in samples from Taiwan (Chan et al., 2022). Another study from Taiwan showed that the use of SGLT2 inhibitors when compared to that of DPP4 inhibitors was associated with a 39% reduction (95% CI, 27%–50%) in the risk of new-onset AF (Ling et al., 2020). A study based in Hong Kong found that SGLT2 inhibitors were associated with a lower risk of new-onset AF (HR 0.68; 95% CI 0.56–0.83) (Lee et al., 2022). Nevertheless, these analyses were conducted among Asian populations, and the results may not be generalizable to those found in other races and ethnicities.

Our current study, conducted in a nationally representative sample of older adults with T2D in Medicare, is one of the first to investigate the effectiveness of SGLT2 inhibitors in reducing the risk of incident AF in the US population and contributes to a better understanding of the potential benefits of SGLT2 inhibitors in AF risk reduction. These findings provide clinicians with valuable guidance for optimizing treatment strategies in individuals with T2D, potentially leading to improved cardiovascular outcomes. Moreover, the observed superiority of SGLT2 inhibitors may translate into tangible healthcare cost savings by reducing diabetes-related complications and healthcare utilization. Our findings also have the potential to influence clinical practice guidelines, prompting updates that prioritize the use of SGLT2 inhibitors for AF prevention in diabetic populations.

Our subgroup analyses revealed the heterogeneity in the effectiveness of SGLT2 inhibitors in reducing AF risk. Specifically, we found that the effect of SGLT2 inhibitors on AF risk reduction remained significant among individuals with established ASCVD and CKD, who are the most vulnerable populations to AF. These results added to the growing evidence suggesting the favorable safety profile of SGLT2 inhibitors and aligned with previous findings of cardiorenal benefits of SGLT2 inhibitors among individuals with established ASCVD and CKD (Toyama et al., 2019; Bhattarai et al., 2022). These results not only emphasize their effectiveness but also support the use of SGLT2 inhibitors as preferred antidiabetic therapies among individuals with specific comorbidities or risk profiles. The observed diverse effects highlight the potential for personalized treatment approaches in diabetic patients with varying comorbidities or risk profiles. By understanding the specific benefits of SGLT2 inhibitors in certain patient populations, clinicians can refine overall treatment strategies and improve the quality of care for individuals with diabetes. Overall, these findings highlight the importance of tailored treatment strategies and represent a significant advancement in diabetes care, shaping clinical decision-making to improve patient outcomes.

We also observed that the protective impact of SGLT2 inhibitors on AF appeared to be profound in non-Hispanic White individuals, but not in other racial and ethnic groups. This heterogeneity could stem from variations in genetic profiles and cardiometabolic risk factors across different racial and ethnic groups. Previous studies have shown that non-Hispanic Black and Hispanic individuals have higher levels of hemoglobin A1c (Smalls et al., 2020) and blood pressure (Redmond et al., 2011), which could explain their distinct response to SGLT2 inhibitors relative to non-Hispanic White individuals (Montvida et al., 2020). Despite this, our study demonstrated that the point estimate for the effect of SGLT2 inhibitors was comparable among non-Hispanic White, non-Hispanic Black, and Hispanic subgroups, but the wider confidence intervals in the latter two groups suggest that the sample size may be insufficient to detect a significant difference. Further research with larger sample sizes or targeting racial minority subgroups is warranted to validate our findings and confirm the effect of SGLT2 inhibitor therapy in AF prevention across various races and ethnicities.

It remains unclear whether AF protection benefits that we observed apply to all SGLT2 inhibitors or if there are significant differences between specific drugs. A meta-analysis by Li et al. indicated that dapagliflozin is associated with a much more obvious risk reduction than empagliflozin and canagliflozin (Li et al., 2022). Conversely, an observational study evaluating the effect of SGLT2 inhibitors on AF risk found that dapagliflozin and empagliflozin offer considerable protective effects (Ling et al., 2020). The reasons behind these potential discrepancies are likely complex and could be linked to the drugs’ differing effects on blood pressure reduction, cardiac remodeling, and metabolic parameters. These results warrant further investigation to confirm the findings and understand any underlying mechanisms for potential differences.

While our retrospective cohort analysis provides valuable insights into the association of SGLT2 inhibitors to the risk of AF when compared with DPP4 inhibitors in Medicare beneficiaries, several limitations must be acknowledged to contextualize our findings. First, our analysis relies on Medicare claims data, which may be subject to incomplete characterization of potentially important covariates. For example, we cannot observe whether a drug filled was taken by patients, as directed. There was no information on clinical exams and laboratory tests such as hemoglobin A1C and body mass index, which may be associated with the severity of diabetes and could have affected the choices of medication. Our analysis did not account for lifestyle factors such as smoking and alcohol consumption, which are known to impact both the risk of AF and the management of T2D (Park et al., 2021). This is due to the fact that claims data only contain information on indirect indicators for lifestyle factors (Brookhart et al., 2010), limiting our ability to account for their influence. However, it is important to consider that the absence of these variables is unlikely to have a significant impact on our findings due to their underrepresentation being non-differential due to the systematic under-coding across the dataset. Another limitation of our study is the dataset’s inconsistency in capturing dosage information, which precluded a robust analysis of the dose-dependent effects. It has been shown that SGLT2 inhibitors exhibit a dose-dependent effect of glycosuria on glucose level, body weight, and blood pressure (Shi et al., 2021). Theoretically, this dose–response relationship could extend to cardiovascular benefits, including prevention of atrial fibrillation, with the hypothesis supported by existing evidence (Zou et al., 2022). Finally, our cohort represents the large population of US Medicare fee-for-service beneficiaries with T2D. The generalizability of our findings to other populations is unknown, such as commercial insurance beneficiaries and Medicaid beneficiaries.

In conclusion, using data from nationally representative samples of Medicare beneficiaries, we identified that the use of SGLT2 inhibitors compared with the use of DPP4 inhibitors was associated with a lower risk of AF. The association was significant in men, non-Hispanic White individuals, and those with established ASCVD and CKD. Our results add valuable insights into the impact of SGLT2 inhibitors on AF in real-world settings and underscore the importance of considering individual characteristics in clinical decision-making regarding GLD for T2D patients, with the aim of optimizing treatment outcomes and minimizing the risk of complications. Further research is needed for exploring the dose-specific effect and the underlying mechanisms of individual SGLT2 inhibitors, comparing their efficacy with other potentially AF-protective medications, and validating the long-term benefit of SGLT2 inhibitors in diverse populations. These efforts are crucial for developing more precise and effective treatment strategies tailored to individuals with type 2 diabetes.

## Data Availability

The data analyzed in this study are subject to the following licenses/restrictions: The Medicare claims data contain patient-level health information and are considered identifiable files. Therefore, access to these data requires a data use agreement. Requests to access these datasets should be directed to resdac@umn.edu.

## References

[B1] American Diabetes Association (2021). 9. Pharmacologic approaches to glycemic treatment: standards of medical care in diabetes—2021. Diabetes Care 44 (1), S111–S124. 10.2337/dc21-s009 33298420

[B2] Arow M. Waldman M. Yadin D. Nudelman V. Shainberg A. Abraham N. G. (2020). Sodium–glucose cotransporter 2 inhibitor Dapagliflozin attenuates diabetic cardiomyopathy. Cardiovasc Diabetol. 19 (1), 7. 10.1186/s12933-019-0980-4 31924211 PMC6953156

[B3] Austin P. C. (2009). Balance diagnostics for comparing the distribution of baseline covariates between treatment groups in propensity-score matched samples. Stat. Med. 28 (25), 3083–3107. 10.1002/sim.3697 19757444 PMC3472075

[B4] Austin P. C. Stuart E. A. (2015). Moving towards best practice when using inverse probability of treatment weighting (IPTW) using the propensity score to estimate causal treatment effects in observational studies. Stat. Med. 34 (28), 3661–3679. 10.1002/sim.6607 26238958 PMC4626409

[B5] Bell D. S. H. Goncalves E. (2019). Atrial fibrillation and type 2 diabetes: prevalence, etiology, pathophysiology and effect of anti-diabetic therapies. Diabetes Obes. Metab. 21 (2), 210–217. 10.1111/dom.13512 30144274

[B6] Bhattarai M. Salih M. Regmi M. Al-Akchar M. Deshpande R. Niaz Z. (2022). Association of sodium-glucose cotransporter 2 inhibitors with cardiovascular outcomes in patients with type 2 diabetes and other risk factors for cardiovascular disease: a meta-analysis. JAMA Netw. Open 5 (1), e2142078. 10.1001/jamanetworkopen.2021.42078 34985519 PMC8733833

[B7] Bolinder J. Ljunggren Ö. Kullberg J. Johansson L. Wilding J. Langkilde A. M. (2012). Effects of dapagliflozin on body weight, total fat mass, and regional adipose tissue distribution in patients with type 2 diabetes mellitus with inadequate glycemic control on metformin. J. Clin. Endocrinol. Metab. 97 (3), 1020–1031. 10.1210/jc.2011-2260 22238392

[B8] Brookhart M. A. Stürmer T. Glynn R. J. Rassen J. Schneeweiss S. (2010). Confounding control in healthcare database research: challenges and potential approaches. Med. Care 48 (6), S114–S120. 10.1097/MLR.0b013e3181dbebe3 20473199 PMC4024462

[B9] Chan Y. H. Chao T. F. Chen S. W. Lee H. F. Li P. R. Chen W. M. (2022). The risk of incident atrial fibrillation in patients with type 2 diabetes treated with sodium glucose cotransporter-2 inhibitors, glucagon-like peptide-1 receptor agonists, and dipeptidyl peptidase-4 inhibitors: a nationwide cohort study. Cardiovasc Diabetol. 21 (1), 118. 10.1186/s12933-022-01549-x 35765074 PMC9241240

[B10] Chronic conditions data warehouse (2022a). Chronic conditions data warehouse. Available at: https://www2.ccwdata.org/condition-categories-chronic (Accessed September 29, 2022).

[B11] Chronic conditions data warehouse (2022b). Chronic conditions data warehouse Medicare administrative data user guide. Available at: https://www2.ccwdata.org/user-documentation (Accessed September 29, 2022).

[B12] Cowie M. R. Fisher M. (2020). SGLT2 inhibitors: mechanisms of cardiovascular benefit beyond glycaemic control. Nat. Rev. Cardiol. 17 (12), 761–772. 10.1038/s41569-020-0406-8 32665641

[B13] Green J. B. Bethel M. A. Armstrong P. W. Buse J. B. Engel S. S. Garg J. (2015). Effect of sitagliptin on cardiovascular outcomes in type 2 diabetes. N. Engl. J. Med. 373 (3), 232–242. 10.1056/NEJMoa1501352 26052984

[B14] Herat L. Y. Magno A. L. Rudnicka C. Hricova J. Carnagarin R. Ward N. C. (2020). SGLT2 inhibitor-induced sympathoinhibition: a novel mechanism for cardiorenal protection. JACC Basic Transl. Sci. 5 (2), 169–179. 10.1016/j.jacbts.2019.11.007 32140623 PMC7046513

[B15] Karayiannides S. Lundman P. Friberg L. Norhammar A. (2018). High overall cardiovascular risk and mortality in patients with atrial fibrillation and diabetes: a nationwide report. Diab Vasc. Dis. Res. 15 (1), 31–38. 10.1177/1479164117735013 29052435

[B16] Kim Y. G. Han K. D. Choi J. I. Boo K. Y. Kim D. Y. Oh S. K. (2019). The impact of body weight and diabetes on new-onset atrial fibrillation: a nationwide population based study. Cardiovasc Diabetol. 18 (1), 128. 10.1186/s12933-019-0932-z 31575379 PMC6774211

[B17] Lee S. Zhou J. Leung K. S. K. Wai A. K. C. Jeevaratnam K. King E. (2022). Comparison of sodium-glucose cotransporter-2 inhibitor and dipeptidyl peptidase-4 inhibitor on the risks of new-onset atrial fibrillation, stroke and mortality in diabetic patients: a propensity score-matched study in Hong Kong. Cardiovasc Drugs Ther. 10, 561–569. Published online February. 10.1007/s10557-022-07319-x PMC10164005 35142921

[B18] Li W. Chen X. Xie X. Xu M. Xu L. Liu P. (2022). Comparison of sodium-glucose cotransporter 2 inhibitors and glucagon-like peptide receptor agonists for atrial fibrillation in type 2 diabetes mellitus: systematic review with network meta-analysis of randomized controlled trials. J. Cardiovasc Pharmacol. 79 (3), 281–288. 10.1097/FJC.0000000000001197 34935705

[B19] Ling A. W. C. Chan C. C. Chen S. W. Kao Y. W. Huang C. Y. Chan Y. H. (2020). The risk of new-onset atrial fibrillation in patients with type 2 diabetes mellitus treated with sodium glucose cotransporter 2 inhibitors versus dipeptidyl peptidase-4 inhibitors. Cardiovasc Diabetol. 19 (1), 188. 10.1186/s12933-020-01162-w 33158436 PMC7648323

[B20] McGuire D. K. Shih W. J. Cosentino F. Charbonnel B. Cherney D. Z. I. Dagogo-Jack S. (2021). Association of SGLT2 inhibitors with cardiovascular and kidney outcomes in patients with type 2 diabetes: a meta-analysis. JAMA Cardiol. 6 (2), 148–158. 10.1001/jamacardio.2020.4511 33031522 PMC7542529

[B21] Montvida O. Verma S. Shaw J. E. Paul S. K. (2020). Cardiometabolic risk factor control in black and white people in the United States initiating sodium-glucose co-transporter-2 inhibitors: a real-world study. Diabetes Obes. Metab. 22 (12), 2384–2397. 10.1111/dom.14164 32744394

[B22] Neal B. Perkovic V. Mahaffey K. W. de Zeeuw D. Fulcher G. Erondu N. (2017). Canagliflozin and cardiovascular and renal events in type 2 diabetes. N. Engl. J. Med. 377 (7), 644–657. 10.1056/NEJMoa1611925 28605608

[B23] Park C. S. Han K. D. Choi E. K. Kim D. H. Lee H. J. Lee S. R. (2021). Lifestyle is associated with atrial fibrillation development in patients with type 2 diabetes mellitus. Sci. Rep. 11 (1), 4676. 10.1038/s41598-021-84307-5 33633333 PMC7907194

[B24] Patorno E. Gopalakrishnan C. Franklin J. M. Brodovicz K. G. Masso-Gonzalez E. Bartels D. B. (2018). Claims-based studies of oral glucose-lowering medications can achieve balance in critical clinical variables only observed in electronic health records. Diabetes Obes. Metab. 20 (4), 974–984. 10.1111/dom.13184 29206336 PMC6207375

[B25] Persson F. Nyström T. Jørgensen M. E. Carstensen B. Gulseth H. L. Thuresson M. (2018). Dapagliflozin is associated with lower risk of cardiovascular events and all-cause mortality in people with type 2 diabetes (CVD-REAL Nordic) when compared with dipeptidyl peptidase-4 inhibitor therapy: a multinational observational study. Diabetes Obes. Metab. 20 (2), 344–351. 10.1111/dom.13077 28771923 PMC5811811

[B26] Redmond N. Baer H. J. Hicks L. S. (2011). Health behaviors and racial disparity in blood pressure control in the national health and nutrition examination survey. Hypertension 57 (3), 383–389. 10.1161/HYPERTENSIONAHA.110.161950 21300667 PMC3048351

[B27] Schotten U. Verheule S. Kirchhof P. Goette A. (2011). Pathophysiological mechanisms of atrial fibrillation: a translational appraisal. Physiol. Rev. 91 (1), 265–325. 10.1152/physrev.00031.2009 21248168

[B28] Scirica B. M. Bhatt D. L. Braunwald E. Steg P. G. Davidson J. Hirshberg B. (2013). Saxagliptin and cardiovascular outcomes in patients with type 2 diabetes mellitus. N. Engl. J. Med. 369 (14), 1317–1326. 10.1056/NEJMoa1307684 23992601

[B29] Shi F. Li H. Shen L. Fu J. J. Ma J. Gu Z. C. (2021). High-dose sodium-glucose co-transporter-2 inhibitors are superior in type 2 diabetes: a meta-analysis of randomized clinical trials. Diabetes Obes. Metab. 23 (9), 2125–2136. 10.1111/dom.14452 34048142

[B30] Shyangdan D. S. Uthman O. A. Waugh N. (2016). SGLT-2 receptor inhibitors for treating patients with type 2 diabetes mellitus: a systematic review and network meta-analysis. BMJ Open 6 (2), e009417. 10.1136/bmjopen-2015-009417 PMC4769433 26911584

[B31] Smalls B. L. Ritchwood T. D. Bishu K. G. Egede L. E. (2020). Racial/ethnic differences in glycemic control in older adults with type 2 diabetes: United States 2003–2014. Int. J. Environ. Res. Public Health 17 (3), 950. 10.3390/ijerph17030950 32033032 PMC7036954

[B32] Stürmer T. Rothman K. J. Avorn J. Glynn R. J. (2010). Treatment effects in the presence of unmeasured confounding: dealing with observations in the tails of the propensity score distribution—a simulation study. Am. J. Epidemiol. 172 (7), 843–854. 10.1093/aje/kwq198 20716704 PMC3025652

[B33] Toyama T. Neuen B. L. Jun M. Ohkuma T. Neal B. Jardine M. J. (2019). Effect of SGLT2 inhibitors on cardiovascular, renal and safety outcomes in patients with type 2 diabetes mellitus and chronic kidney disease: a systematic review and meta-analysis. Diabetes Obes. Metab. 21 (5), 1237–1250. 10.1111/dom.13648 30697905

[B34] Wang A. Green J. B. Halperin J. L. Piccini J. P. (2019). Atrial fibrillation and diabetes mellitus: JACC review topic of the week. J. Am. Coll. Cardiol. 74 (8), 1107–1115. 10.1016/j.jacc.2019.07.020 31439220

[B35] Yao R. J. R. Andrade J. G. Deyell M. W. Jackson H. McAlister F. A. Hawkins N. M. (2019). Sensitivity, specificity, positive and negative predictive values of identifying atrial fibrillation using administrative data: a systematic review and meta-analysis. Clin. Epidemiol. 11, 753–767. 10.2147/CLEP.S206267 31933524 PMC6712502

[B36] Zelniker T. A. Bonaca M. P. Furtado R. H. M. Mosenzon O. Kuder J. F. Murphy S. A. (2020). Effect of dapagliflozin on atrial fibrillation in patients with type 2 diabetes mellitus: insights from the DECLARE-TIMI 58 trial. Circulation 141 (15), 1227–1234. 10.1161/CIRCULATIONAHA.119.044183 31983236

[B37] Zinman B. Wanner C. Lachin J. M. Fitchett D. Bluhmki E. Hantel S. (2015). Empagliflozin, cardiovascular outcomes, and mortality in type 2 diabetes. N. Engl. J. Med. 373 (22), 2117–2128. 10.1056/NEJMoa1504720 26378978

[B38] Zou H. T. Yang G. H. Cai Y. J. Chen H. Zheng X. Q. Hu R. (2022). Are high- or low-dose SGLT2 inhibitors associated with cardiovascular and respiratory adverse events? A meta-analysis. J. Cardiovasc Pharmacol. 79 (5), 655–662. 10.1097/FJC.0000000000001222 35058411

