# Supplementary material for: Sodium-glucose cotransporter-2 inhibitors and incidence of atrial fibrillation in older adults with type 2 diabetes: a retrospective cohort analysis
Source: Front Pharmacol. 2024 May 23;15:1379251. doi: 10.3389/fphar.2024.1379251 (PMC11153786; doi:10.3389/fphar.2024.1379251)
Supplement: Supplementary file 1 [file DataSheet1.docx]

Supplementary Material

**Sodium-glucose Cotransporter-2 Inhibitors and Incidence of Atrial Fibrillation in Older Adults with Type 2 Diabetes: A Retrospective Cohort Analysis**

Yujia Li, Huilin Tang, Yi Guo, Hui Shao, Stephen E. Kimmel, Jiang Bian, Desmond A. Schatz, Jingchuan Guo

**Supplementary Figure 1.** Cumulative incidence function of atrial fibrillation comparing type 2 diabetes patients treated with a sodium glucose cotransporter 2 inhibitor (SGLT2i) versus those treated with a dipeptidyl peptidase-4 inhibitors (DPP4i) after inverse probability of treatment weighting (IPTW).

**Supplementary Table 1.** Summary of the effect of SGLT-2 inhibitors on atrial fibrillation outcome compared with DPP-4 inhibitors in subgroups stratifying by patients by concomitant use with other glucose-lowering drugs.


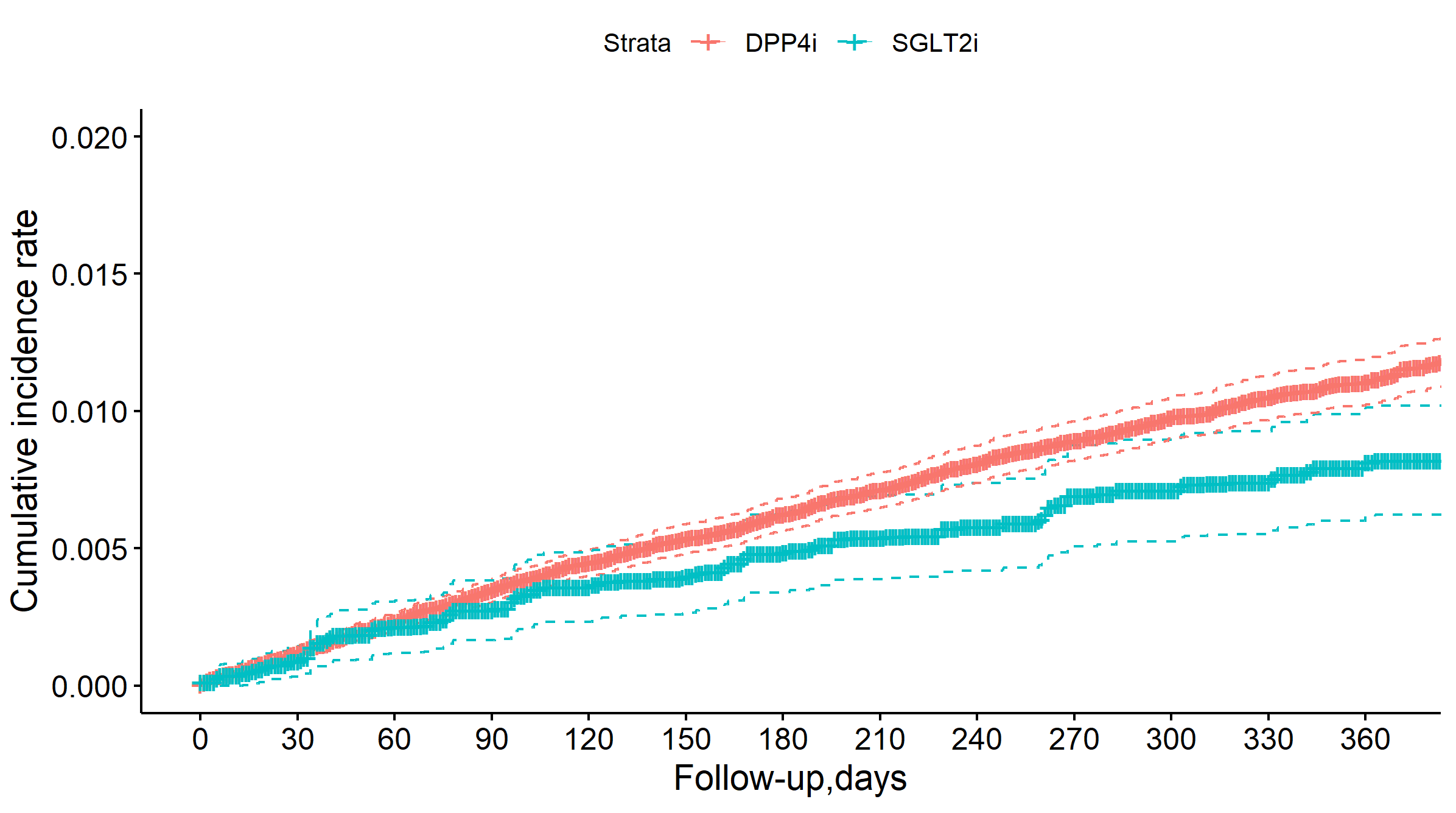


**Supplementary Figure 1.** Cumulative incidence function of atrial fibrillation comparing type 2 diabetes patients treated with a sodium glucose cotransporter 2 inhibitor (SGLT2i) versus those treated with a dipeptidyl peptidase-4 inhibitors (DPP4i) after inverse probability of treatment weighting (IPTW).

**Supplementary Table 1.** Summary of the effect of SGLT-2 inhibitors on atrial fibrillation outcome compared with DPP-4 inhibitors in subgroups stratified patients by concomitant use with other glucose-lowering drugs. PY, person-years; HR, hazard ratio; CI, confidence interval; GLP1, glucagon-like peptide-1.

|  | Total, n | Cases, n (%) | Crude incidence rate,  per 1000 PY | HR (95% CI) | p-value |
| --- | --- | --- | --- | --- | --- |
| Overall | 97436 | 983 (1.01) | 11.24 (10.57, 11.97) | 0.727 (0.567, 0.907) | 0.01 |
| Metformin |  |  |  |  |  |
| No | 53200 | 637 (1.20) | 13.43 (12.43, 14.50) | 0.961 (0.695, 1.330) | 0.81 |
| Yes | 44236 | 346 (0.78) | 8.64 (7.78, 9.60) | 0.836 (0.611, 1.145) | 0.26 |
| Thiazolidinediones |  |  |  |  |  |
| No | 89815 | 929 (1.03) | 11.50 (10.79, 12.26) | 0.717 (0.564, 0.913) | <0.01 |
| Yes | 7621 | 54 (0.71) | 8.04 (6.17, 10.49) | 0.629 (0.208, 1.898) | 0.41 |
| Sulfonylureas |  |  |  |  |  |
| No | 60837 | 597 (0.98) | 10.96 (10.12, 11.87) | 0.700 (0.518, 0.947) | 0.02 |
| Yes | 36599 | 386 (1.05) | 11.69 (10.59, 12.91) | 0.833 (0.572, 1.212) | 0.34 |
| GLP-1 receptor agonists |  |  |  |  |  |
| No | 91328 | 921 (1.01) | 11.23 (10.53, 11.97) | 0.780 (0.604, 1.007) | 0.06 |
| Yes | 6108 | 62 (1.02) | 11.32 (8.84, 14.51) | 0.858 (0.499, 1.474) | 0.58 |
